# A Sensitive and Selective Electrochemical Aptasensor for Carbendazim Detection

**DOI:** 10.3390/bios15010015

**Published:** 2025-01-03

**Authors:** Suthira Pushparajah, Mahnaz Shafiei, Aimin Yu

**Affiliations:** School of Science, Computing, and Engineering Technology, Swinburne University of Technology, Hawthorn, VIC 3122, Australia; spushparajah@swin.edu.au

**Keywords:** electrochemical aptasensor, carbendazim, reduced graphene oxide, platinum nanoparticles

## Abstract

Carbendazim (CBZ) is used to prevent fungal infections in agricultural crops. Given its high persistence and potential for long-term health effects, it is crucial to quickly identify pesticide residues in food and the environment in order to mitigate excessive exposure. Aptamer-based sensors offer a promising solution for pesticide detection due to their exceptional selectivity, design versatility, ease of use, and affordability. Herein, we report the development of an electrochemical aptasensor for CBZ detection. The sensor was fabricated through a one-step electrodeposition of platinum nanoparticles (Pt NPs) and reduced graphene oxide (rGO) on a glassy carbon electrode (GCE). Then, a CBZ-specific aptamer was attached via Pt-sulfur bonds. Upon combining CBZ with the aptamer on the electrode surface, the redox reaction of the electrochemical probe K_4_[Fe(CN)_6_] is hindered, resulting in a current drop. Under optimized conditions (pH of 7.5 and 25 min of incubation time), the proposed aptasensor showed a linear current reduction to CBZ concentrations between 0.5 and 15 nM. The limit of detection (LOD) for this proposed aptasensor is 0.41 nM. Along with its repeatable character, the aptasensor demonstrated better selectivity for CBZ compared to other potential compounds. The recovery rates for detecting CBZ in skim milk and tap water using the standard addition method were 98% and 96%, respectively. The proposed aptasensor demonstrated simplicity, sensitivity, and selectivity for detecting CBZ with satisfactory repeatability. It establishes a strong foundation for environmental monitoring of CBZ.

## 1. Introduction

Carbendazim (CBZ) is a systemic benzimidazole fungicide that is often used in agriculture. CBZ is one of the most persistent environmental agents due to its slow degradation, limited solubility in water, and extended half-life [1]. Fruits, vegetables, and other food products have been shown to contain CBZ residues, thus impacting consumer safety. As a result, monitoring residual CBZ in agricultural products is important. Various toxicological investigations have shown that a certain dosage of CBZ may lead to carcinogenic and teratogenic impacts in humans and animals [2]. After its negative effects were discovered, several nations implemented strict maximum residue limits for CBZ in food and other agricultural products. For example, China has established maximum residue limits for CBZ in apples, which are reported as 5 mg/kg. In contrast, CBZ is strictly banned in the USA and Australia [3]. Therefore, quick, reliable, and sensitive CBZ detection techniques are important to ensure health and environmental protection.

Common detection methods, such as liquid chromatography-tandem mass spectrometry (LC-MS), gas chromatography-tandem mass spectrometry (GC-MS) [4], high-performance liquid chromatography (HPLC) [5], colorimetry [6], fluorometry [7], and surface-enhanced Raman spectroscopy [8] offer high sensitivity and reliability. The remarkable sensitivity and precision of HPLC have made it the principal analytical tool for detecting and measuring pesticides. For example, the HPLC-DAD (diode array detector) technique is used to detect CBZ [5] with 0.45 µg/L of the limit of detection (LOD). While the above approaches are very sensitive and reliable, they also have drawbacks, including the need for expensive instruments, complicated pretreatment stages, time-consuming detection processes, and the necessity for trained staff [9]. These methods have limited practical applications in real-time pesticide residue detection.

Aptamers are synthesized from single-stranded DNA or RNA molecules. Through the interaction of its nucleobases, aptamers can fold into a 3D structure that conforms to the shape of the target molecule. Aptamers can interact with their target molecules via hydrogen bonding, van der Waals forces, and electrostatic interactions. There have been some reports on CBZ detection using electrochemical aptasensors. For instance, an electrochemical aptasensor employing carbon nanotubes for CBZ detection was developed by Venegas et al. [10]. A carbodiimide technique was used to covalently immobilize an amino-terminated aptamer with carboxylic groups of carbon nanotubes. When CBZ was found, the voltammetric response was reduced, suggesting a change in aptamer conformation that prevented the redox probe from reaching its target. Under ideal conditions, the proposed aptasensor recorded an LOD of 4.35 nM. Zourob developed a CBZ aptasensor using a self-assembly approach to immobilize a thiol-modified aptamer on a gold electrode [11]. The LOD for this suggested aptasensor was 0.05 nM. Wang et al. later designed another aptasensor using a combination of gold nanoparticles (Au NPs) and boron nitride nanocrystals [12]. A methylene blue-labeled single-strand oligonucleotide was attached to a glassy carbon electrode (GCE) to facilitate aptamer hybridization. The LOD of the proposed aptasensor was 0.099 nM. Furthermore, an impedance-based aptasensor was designed by Zhu et al. to detect CBZ [13]. The thiolated aptamer was bound to a composite of carbon nanohorns and Au NPs via the Au-S bond. The LOD for this suggested aptasensor was 0.003 nM for CBZ detection. In addition to that, sensitivity was further improved by adding advanced nanomaterials and different sensing techniques. For instance, Jin et al. proposed an aptasensor using mulberry fruit-like gold nanocrystals and multiple graphene aerogels using a DNA cycle amplification strategy [14]. The LOD of the developed aptasensor was 0.044 fM for CBZ detection. Similarly, Khosropour et al. developed an impedance-based aptasensor using graphene nanoribbons, Au NPs, and a metallic organic framework to detect CBZ [15]. This aptasensor was performed based on the double signal amplification strategy, which recorded an LOD of 0.4 fM.

**Figure 1:**
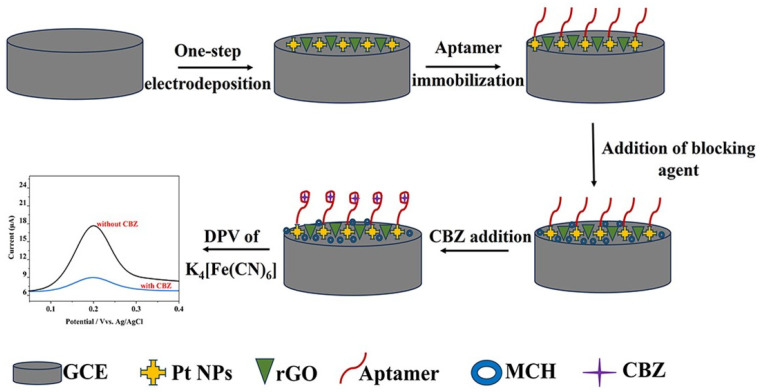
Schematic illustration of the preparation of the electrochemical aptasensor for CBZ detection.

The proposed research aims to improve the selectivity and sensitivity of CBZ detection by integrating platinum nanoparticles (Pt NPs) and reduced graphene oxide (rGO) with a CBZ-specific aptamer. Pt NPs are known for their high catalytic characteristics and large surface-area-to-volume ratio, but Pt NPs are susceptible to substantial agglomeration, leading to an adverse impact on their catalytic efficiency. It was reported that depositing Pt NPs onto carbon substrates, such as graphene and its derivatives, could improve dispersibility [16]. Additionally, rGO can improve electrochemical signals through its large surface area and high electrical conductivity [17]. A previous work proposed an electrochemical aptasensor design that includes a one-step electrodeposition of Pt NPs and rGO onto a GCE surface [18]. In this study, the thiol-modified aptamer was attached to the Pt-rGO/GCE surface through a Pt-sulfur bond (Figure 1). The study applied cyclic voltammetry (CV), differential pulse voltammetry (DPV), and electrochemical impedance spectroscopy (EIS) to investigate the electrochemical performance of the sensor. This work aims to develop a highly sensitive and selective CBZ-specific aptasensor with excellent repeatability.

## 2. Materials and Methods

### 2.1. Materials

All chemicals were analytical-grade and were used without additional purification. Platinum (II) chloride (metal basis 98%, PtCl_2_), carbendazim (CBZ, 97%), potassium hexacyanoferrate (II) trihydrate (K_4_[Fe(CN)_6_·3H_2_O] ), potassium iodide (KI), ammonium sulfate (NH_4_)_2_SO_4_, sodium chloride (NaCl), dibasic sodium phosphate (Na_2_HPO_4_), monobasic sodium phosphate (NaH_2_PO_4_), tris (hydroxymethyl) aminomethane hydrochloride salt (Tris-HCl), sodium dodecyl sulfate (SDS), potassium nitrate (KNO_3_), and tween 20 were purchased from Sigma-Aldrich (Darmstadt, Germany). Tris-(2-carboxyethyl) phosphine hydrochloride (TCEP) (≥98%) and 6-Mercapto-1-hexanol (MCH) (>98%) were purchased from Chem Supply Australia Pty. Ltd. (Port Adelaide, Australia). Graphene oxide (GO) powder was obtained from JCNANO, Inc. (Nanjing, China). Hydrochloric acid (HCl), sodium hydroxide (NaOH) pellets, and D-glucose (AR grade) were acquired from Merck Pty Ltd. (Darmstadt, Germany). Ethylenediaminetetraacetic acid disodium salt dihydrate (EDTA) was obtained from Thermo Fisher Scientific Australia Pty, Ltd. (City of Knox, Australia). A glassy carbon electrode (GCE, 3 mm in diameter), Ag/AgCl (3 M KCl) electrode, platinum wire electrode, and gamma alumina powder (1.0, 0.3, 0.05 μm) were acquired from Gaoss Union Company (Wuhan, China). Phosphate buffer solutions (PBS) were prepared using a combination of 0.1 M Na_2_HPO_4_ and NaH_2_PO_4_. A solution containing 0.1 M HCl and 0.1 M NaOH was used to adjust the pH of the mixture. Milli-Q water (18.2 MΩ cm) was used in all studies. An aptamer specific to CBZ was synthesized by Sangon Biotechnology Co., Ltd. (Shanghai, China). The thiol-modified sequence was 5′-C6/SH/GGG CAC ACA ACA ACC GAT GGT CCA GCC ACC CGA ATG ACC AGC CCA CCC GCC ACC CCG CG/3′ [11]. Tris-HCl buffer (Tris (10 mM) and EDTA (0.1 mM)) (pH 7.4 ± 7.6) was used for the aptamer stock solutions and resuspension.

### 2.2. Preparation of Aptasensors for CBZ Detection

An ultrasonic bath was used to clean the GCE for 3 min before electrodeposition. The GCE was polished using 1 µm of alumina particles and cleaned with water using a 3-min ultrasonic technique. The process was carried out again, applying alumina particles that were 0.3 µm and 0.05 µm in size, respectively. Subsequently, the polished electrodes were subjected to two ultrasonic treatments with water and 100% ethanol, each lasting three min. The cleaned GCE was immersed in a mixed solution of PtCl_2_ solution (0.5 mg/mL) and GO solution (1.0 mg/mL). The electrodeposition procedure was conducted using the CV method at a scan rate of 0.1 V/s. The potential scan was repeatedly cycled from −1.5 to 1.0 V for 20 cycles. During this process, GO and Pt^2+^ ions were simultaneously electrochemically reduced onto the electrode surface. After electrodeposition, the Pt-rGO/GCE was rinsed with water and air-dried. Subsequently, thiol-modified aptamers (10 µM) were activated by reducing disulfide bonds using 1 mM TCEP. The sample was placed in a dark environment at room temperature for 1 h. Then, the aptamer was incubated overnight with Pt-rGO/GCE at 4 °C. This procedure is essential to avoid evaporation of the solution and guarantee the efficient integration of aptamers with the nanomaterial-modified electrode. Following incubation, the Apt-Pt-rGO/GCE was rinsed with 10 mM Tris-HCl solution to eliminate any unbound sequence on the aptasensor surface and allowed to dry. The Apt-Pt-rGO/GCE was incubated with 1 mM of MCH at room temperature for 1 h to inhibit any residual active sites and prevent the existence of non-specific adsorption on the electrode surface. After washing with 10 mM Tris-HCl solution to remove any remaining MCH molecules, the prepared Apt-Pt-rGO/GCE was preserved at 4 °C for future use.

### 2.3. Electrochemical Measurements

The electrochemical experiments were conducted using a CHI660 electrochemical workstation (Champaign, IL, USA) in a standard three-electrode configuration: a working electrode (bare or modified GCE), a reference electrode (Ag/AgCl (3 M KCl)), and an auxiliary electrode (platinum wire). For the CV and DPV testing, the potential range of −0.3 to 0.8 V was used to measure the current from K_4_[Fe(CN)_6_]. The EIS tests were performed with a bias potential of 0.2 V and frequencies that ranged from 10^−2^ to 10^3^ Hz. For the previously mentioned CV and EIS tests, a solution containing 1.0 mM K_4_[Fe(CN)_6_] in 0.1 M KCl was used. The solution contained 1.0 mM K_4_[Fe(CN)_6_] in 0.1 M KCl and 0.1 M PBS (pH 7.5) used for the DPV experiments. The structural morphology of each modified GCE was analyzed using a ZEISS SUPRA 40 PV scanning electron microscope (SEM) combined with energy-dispersive X-ray spectroscopy (Carl Zeiss NTS Gmbh, Oberkochen, Germany). A Kratos AXIS NOVA spectrometer (Kratos Analytical, Inc., Manchester, UK) was used to assess the elemental composition of the aptamer-modified electrode, and Casa XPS 2.3.19PR1.0 software was used to interpret the results.

### 2.4. Recovery Tests for CBZ

A stock solution of CBZ (500 μM) was prepared by dissolving CBZ powder in water. Skim milk (Coles supermarket in Melbourne, Australia) and tap water were filtered to discard any particles before use. A solution was prepared by combining 1.0 mM K_4_[Fe(CN)_6_] and 0.1 M PBS (pH of 7.5) in a 1:1 ratio with tap water or skim milk. This matrix served as the electrochemical condition for testing before adding an appropriate amount of CBZ stock solution. The trials were conducted at room temperature with three replicates.

## 3. Results and Discussion

### 3.1. Material Characterization

**Figure 2:**
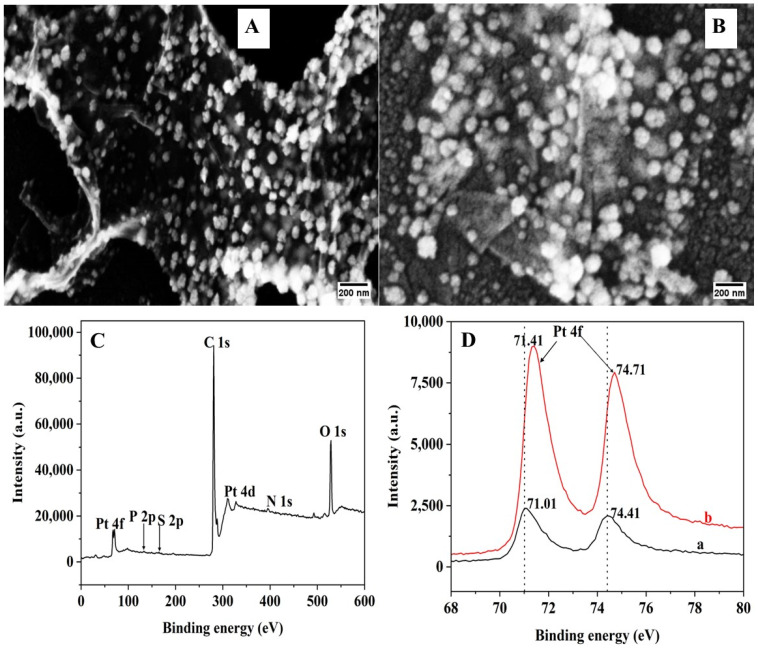
SEM images of (**A**) Pt-rGO/GCE and (**B**) Apt-Pt-rGO/GCE. XPS spectra of (**C**) wide scan of Apt-Pt-rGO/GCE, and (**D**) Peak binding energy shift of Pt 4f (a) before and (b) after aptamer immobilization.

The Pt-rGO-modified electrode was first prepared by concurrently electrodepositing GO and Pt NPs on the GCE using the CV technique. Then, the thiol-modified aptamer was immobilized onto the electrode to obtain Apt-Pt-rGO/GCE. The surface morphology of each modified electrode was analyzed using SEM. GO possesses a distinctive crumpled and wrinkled appearance, as seen in Figure 2A, since it was synthesized via the electrochemical reduction process [19]. Additionally, a large number of smooth, cauliflower-shaped Pt NPs are uniformly scattered around the rGO surface [20]. The average size of the Pt NPs was about 50 nm (Figure S1A). As seen in Figure 2B, the surface morphology noticeably changed with a filmy appearance following aptamer immobilization. Specifically, Pt NPs became weaker and more irregular in shape due to aptamer attachment [21,22]. The average size of the Pt NPs in the aptamer-modified electrode was about 53 nm (Figure S1B).

XPS analysis was applied to confirm the formation of the Pt-rGO nanocomposite and aptamer immobilization. As shown in Figure 2C, the XPS spectra support the deposition of Pt and rGO onto the GCE by displaying the presence of the following peaks: C (1s) at 284.8 eV, O (1s) at 532 eV, Pt (4d) at 71 eV, and Pt (4f) at 314 eV. Further, the increased level of the N (1s) at 399.8 eV (Figure S1C), P (2p) at 130 eV (Figure S1D), and S (2p) at 164 eV (Figure S1E) peak intensities after the immobilization of aptamers provides evidence for aptamer attachment. As shown in Figure 2D(a), the binding energy of 71.01 and 74.41 eV of Pt (4f) at the Pt-rGO/GCE sample shows the presence of Pt in its zero-valent form [23]. The Apt-Pt-rGO/GCE exhibits a peak shift at 71.41 and 74.71 eV, as seen in Figure 2D(b). This peak shift may be attributed to the existence of Pt ^δ+^ species, which are a consequence of the charge transfer from Pt NPs to S atoms [24]. This peak is probably caused by Pt atoms bound to S in the thiol-modified aptamer. This energy is often linked to the thiol group or disulfide compounds [25]. Nevertheless, the measured binding energies align with the findings reported in the literature regarding the interaction between Pt and S [24,26]. The shift in Pt binding energy further confirms that the aptamer is successfully immobilized onto the Pt-rGO/GCE via Pt-S bonds.

### 3.2. Electrochemical Performance

**Figure 3:**
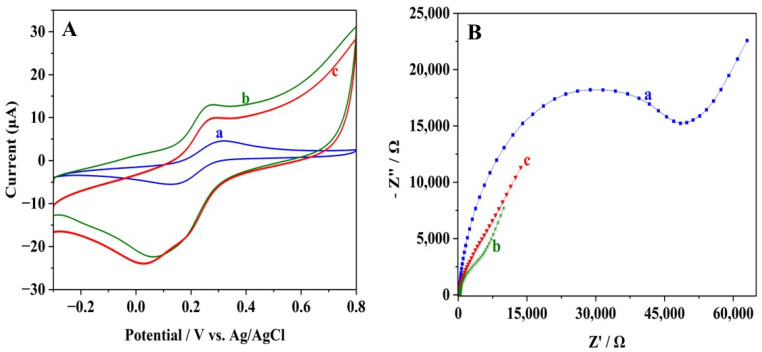
(**A**) CV plots and (**B**) Nyquist diagrams of EIS of (a) bare GCE, (b) Pt-rGO/GCE, and (c) Apt-Pt-rGO/GCE in a 0.1 M KCl solution containing 1.0 mM K_4_[Fe(CN)_6_].

The CV graphs in Figure 3A show the current response of 1.0 mM K_4_[Fe(CN)_6_] at different electrodes measured using a 0.1 M KCl solution containing 1.0 mM K_4_[Fe(CN)_6_]. A pair of reversible redox peaks of [Fe(CN)_6_]^4−^ was observed at the bare GCE, which is shown by curve a. There is a significant peak current increase at the Pt and rGO-modified electrode (Pt-rGO/GCE) (curve b), which is due to the increased effective surface area and enhanced electrical conductivity of the electrode. Nevertheless, immobilization of the CBZ-specific aptamer (Apt-Pt-rGO/GCE) led to a small reduction in the current response (curve c). The main influencing factor is the nature of the aptamer, which is a non-conductive organic molecule. As a result, it hindered the electron transmission from the redox probe [Fe(CN)_6_]^4−^ to the electrode.

The EIS provides further confirmation of the variations in conductivity between the electrodes. The Pt-rGO/GCE exhibits a smaller half-circle diameter (Figure 3B, curve b) compared to the bare GCE (Figure 3B, curve a), showing lower impedance due to the improved electrical conductivity and larger effective surface area. Therefore, the modification of the GCE with Pt-rGO reduces the Ret to approximately 5000 Ω. However, when the aptamer is immobilized, there is an increase in Ret (~6000 Ω), as shown in Figure 3B (curve c), confirming that the aptamer’s organic and non-conductive nature hinders electron transmission from the redox probe to the electrode.

### 3.3. Feasibility of the Aptasensor for CBZ Detection

**Figure 4:**
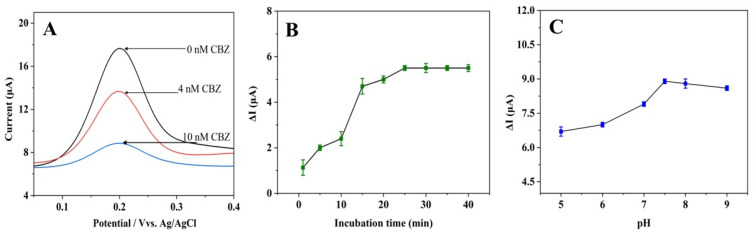
(**A**) DPVs of 1.0 mM K_4_[Fe(CN)_6_] at the aptasensor before and after adding 4 nM and 10 nM of CBZ in pH 7.0 PBS containing 0.1 M KCl. The effects of (**B**) incubation time (pH fixed at 7.0) and (**C**) pH (incubation time fixed at 25 min) on the CBZ current response.

The electrochemical aptasensor was assessed for its feasibility in detecting CBZ. Figure 4A shows the DPV responses of the Apt-Pt-rGO/GCE at various CBZ concentrations (0, 4, and 10 nM). In the absence of CBZ, the electrochemical probe K_4_[Fe(CN)_6_] produced a peak current value of 17.66 µA. Upon addition of CBZ, a corresponding decrease in the peak current was observed in the DPV spectrum. This decrease in current results from the binding of non-conductive CBZ molecules, which impedes electron transmission from the redox probe to the electrode. The findings show that the CBZ binds aptasensor successfully, forming an aptamer-CBZ complex that influences the sensor’s electrochemical response to different CBZ concentrations.

### 3.4. Optimization of Experimental Parameters

The experimental conditions, such as the CBZ incubation time and pH of the buffer for aptasensor fabrication, were optimized to improve the detection performance. The Apt-Pt-rGO/GCE was incubated with CBZ at pH 7 to determine the best incubation time for CBZ detection. Figure 4B illustrates that the current change increased gradually from 1 to 25 min, reaching its maximum and stabilizing thereafter. This result shows that the CBZ-aptamer complex achieved its maximum binding capacity within 25 min [27]. The current stability up to 40 min indicates that the Apt-Pt-rGO/GCE and CBZ have achieved equilibrium with no further reaction. Consequently, an optimal CBZ incubation time of 25 min was chosen for the measurement of CBZ.

Then, the Apt-Pt-rGO/GCE was immersed in 1.0 mM K_4_[Fe(CN)_6_] at different pH levels in PBS with CBZ for 25 min to investigate the optimal pH of the PBS buffer. As shown in Figure 4C, the change in current increased as the pH increased from 5.0 to 7.5, with the change reaching its highest point at pH 7.5. The aptamer likely develops a stable conformation for binding CBZ near neutral pH 7.5, enhancing sensor sensitivity. A neutral pH also reduces interference with both the aptamer and CBZ, enhancing electron transfer. However, at pH levels above 7.5, a gradual decline in the current change was observed. This result suggests that a higher pH may destabilize the aptamer structure, lowering its binding affinity for CBZ [28]. Further, pH changes could alter CBZ’s chemical properties, affecting its interaction with the aptamer and electrochemical medium [29]. Therefore, a pH of 7.5 in PBS buffer was selected for further experiments.

### 3.5. Electrochemical Detection of CBZ

**Figure 5:**
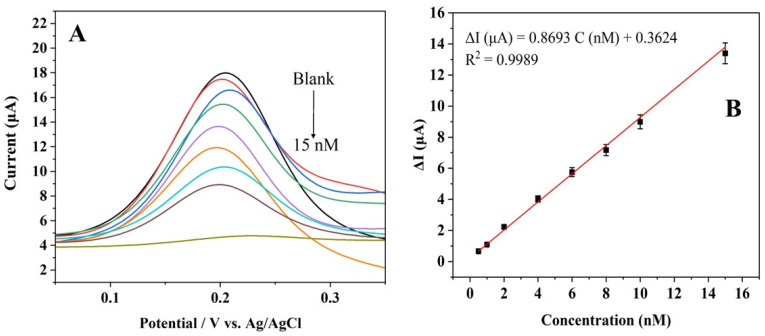
(**A**) DPV responses of the aptasensor toward CBZ with different concentrations (0, 0.5, 1, 2, 4, 6, 8, 10, and 15 nM) in pH 7.5 PBS containing 1.0 mM K_4_[Fe(CN)_6_] and 0.1 M KCl. (**B**) Linear curve of ΔI vs. CBZ concentration (nM).

With optimized experimental conditions, the Apt-Pt-rGO/GCE was applied to detect CBZ at concentrations varying from 0.5 to 15 nM through DPV. Figure 5A shows a continuous decrease in the aptasensor’s current signal as the CBZ concentration increases. The change in peak current (Δ*I*) was calculated from the difference in peak currents before and after CBZ addition. A standard curve was plotted using the peak current change (Δ*I*) versus the concentration of CBZ (nM) (Figure 5B). A positive correlation was observed between the current change and the CBZ concentration. A strong linear relationship was developed using the equation Δ*I* (µA) = 0.8693 C (nM) + 0.3624 (R^2^ = 0.9989). The LOD was determined to be 0.41 nM.

### 3.6. Selectivity, Repeatability, and Stability Characterization

**Figure 6:**
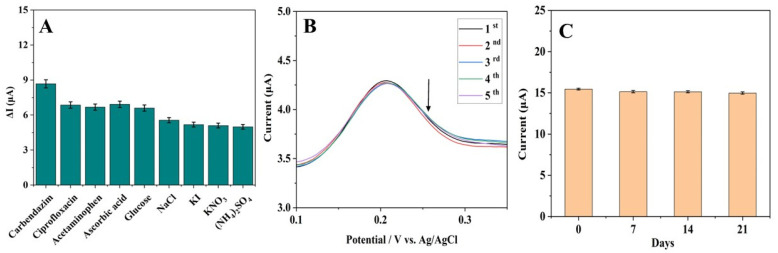
(**A**) Selectivity performance of the aptasensor in 10 nM of ciprofloxacin, acetaminophen, ascorbic acid, glucose, NaCl, KI, KNO_3_, and (NH_4_)_2_SO_4_ in pH 7.5 PBS containing 1.0 mM K_4_[Fe(CN)_6_]. (**B**) Repeatability of the aptasensor in five samples containing 15 nM CBZ. (**C**) Current response of the aptasensor to 2 nM of CBZ when kept at 4 °C for 0, 7, 14, and 21 days.

The aptasensor’s selectivity was investigated by measuring the current response toward CBZ and other potential interferences (ciprofloxacin, acetaminophen, ascorbic acid, glucose, NaCl, KI, KNO_3_, and (NH_4_)_2_SO_4_), each present at a concentration of 10 nM. As shown in Figure 6A, all tested compounds showed a current drop similar to that of CBZ. However, the response to CBZ exhibits the highest current change, indicating that the aptamer has a better recognition of CBZ over other compounds. This result may be due to the better interaction and binding of the designed aptamer to CBZ via hydrogen bonding between functional groups and shape complementarities [30]. Although other compounds with similar functional groups can also bind to aptamers, the interaction between the aptamer and the CBZ functional group was stronger, leading to an increased response. It is also noted that other compounds might attach to the sensor electrode via non-specific adsorption, which could also lead to some changes in the current. One of the primary advantages of aptamers is still their high affinity and specificity for a target, such as CBZ.

To evaluate repeatability, five successive samples containing 15 nM CBZ were quantified using DPV with Apt-Pt-rGO/GCE (Figure 6B). The calculated relative standard deviation (RSD)% of 2.8% shows that the sensor exhibits an acceptable level of repeatability. The stability of the electrochemical aptasensor was tested by measuring the DPV current signal after storage at 4 °C for 0, 7, 14, and 21 days. The 2 nM of CBZ was used to measure the current response at each interval. The current decreased by 1.4% after 7 days, 1.7% after 14 days, and 3.7% after 21 days, as shown in Figure 6C. After 21 days of storage, the peak current remained close to 96% of the original response. These results demonstrate the biosensor’s sufficient stability for future applications.

### 3.7. Regeneration of the Apt-Pt-rGO/GCE Sensor

Aptamers have a strong affinity for their target molecules, making it challenging to remove these attached molecules. Washing with buffers, detergents, water, strong solutions of salts, acids, or bases, and chelating agents like EDTA or urea are common chemical treatments for regeneration purposes. These treatments act by dissociating the aptamer-target complex and breaking the affinity bond between the aptamer and target molecules. Adjusting the pH and temperature could also facilitate the removal of attached target molecules [31,32].

In this study, the regeneration of the aptasensor was tested with 10 mM NaCl (salt), 10 mM NaOH (base), 2% SDS solution (detergent), 40 mM tris-HCl (pH 8.0), and 10 mM EDTA with 0.02% tween-20 (chelating agent). The electrode’s peak current increased significantly after being incubated with 10 mM NaOH (Figure S2A), 40 mM tris-HCl (pH 8.0), and 10 mM EDTA with 0.02% tween-20 (Figure S2B), suggesting that the bound CBZ was successfully removed. After regeneration, the electrodes were tested for CBZ. The peak current was reduced after incubation with 10 µM CBZ, indicating that the regenerated electrode could effectively re-bind CBZ with a 100% regeneration capacity. However, regeneration using 2% SDS and 10 mM NaCl was less effective, as shown in Figure S2C and S2D, respectively. Recent research has shown that NaOH is frequently used for effective regeneration and successfully eliminates different biomolecules, such as bacteria, proteins, and nucleic acids, from biosensor systems [33,34]. For CBZ sensing in further studies, 10 mM NaOH was used to regenerate the electrode.

### 3.8. Recovery Test for CBZ in Skim Milk and Tap Water

The developed aptasensor was tested for CBZ in skim milk and tap water to evaluate its potential for practical applications. A calibration plot (Figure 5B) was used to determine CBZ concentrations. Table 1 shows the recovery results of this investigation. The average CBZ recovery in skim milk was 98%, while it was 96% in tap water. The RSD for skim milk was 2%, and for tap water, it was 1%. These results suggest that the developed electrochemical aptasensor has potential for CBZ analysis in skim milk and tap water.

## 4. Conclusions

A CBZ electrochemical aptasensor was successfully developed by immobilizing a CBZ-specific aptamer on Pt-rGO/GCE through Pt-sulfur bonds. CV, EIS, and XPS were used to investigate the electrochemical characteristics and properties of the Pt NPs, rGO, and aptamer-modified electrodes, respectively. It was confirmed that the aptamer binds to the Pt-rGO/GCE through a Pt-sulfur bond. The addition of CBZ to the Apt-Pt-rGO/GCE further confirmed that the sensor became less conductive in order to reduce the electron transfer rate from the redox probe. The developed aptasensor has the capability to regenerate with 10 mM NaOH. The aptasensor, with its unique receptor, demonstrated better selectivity for CBZ compared to other similar compounds. It also displayed satisfactory repeatability and an LOD of 0.41 nM. The feasibility of the practical application was verified through CBZ recovery in skim milk (98%) and tap water (96%). Hence, this electrochemical aptasensor provides a straightforward and consistent approach for ascertaining the presence of pesticides in food samples and other agricultural products.

## Figures and Tables

**Table 1:** CBZ recovery test in tap water and skim milk (*n* = 3).

Mixture	Added (nM)	Found (nM)	Recovery (%)	RSD (%)
Buffer: skim milk (1:1)	14	13.7	98	3.6
	12	12.0	100	1.0
	3.0	3.1	103	2.1
	1.0	0.9	90	0.9
Buffer: tap water (1:1)	15	15.0	100	0.9
	13	12.0	92	0.6
	3.0	3.0	100	1.8
	1.0	0.9	90	0.7

## Data Availability

The data that support the findings of this study are available from the corresponding authors upon reasonable request.

## Supplementary Materials

The following supporting information can be downloaded at https://www.mdpi.com/article/10.3390/bios15010015/s1, Figure S1. Pt particle size distribution histogram for (A) Pt-rGO/GCE, (B) Apt-Pt-rGO/GCE and (C-E) peak confirmation for N 1s, P 2p, and S 2p in Apt-Pt-rGO/GCE corresponds to XPS results. Figure S2. Regeneration of electrodes with (A) 10 mM NaOH (B) 40 mM tris-HCl (pH 8.0), 10 mM EDTA with 0.02% tween-20 (C) 2% SDS solution, and (D) 10 mM NaCl salt. Curve a indicates the current before incubation, curve b indicates the current after incubation with regeneration agents, and curve c indicates the current with 10 µM CBZ.
