# Supplementary material for: A Sensitive and Selective Electrochemical Aptasensor for Carbendazim Detection
Source: Biosensors (Basel). 2025 Jan 3;15(1):15. doi: 10.3390/bios15010015 (PMC11764140; doi:10.3390/bios15010015)
Supplement: Supplementary file 1 [file biosensors-15-00015-s001.zip › biosensors-3380235-supplementary.pdf]

Supporting Information

A Sensitive and Selective Electrochemical Aptasensor for Carbendazim Detection

Suthira Pushparajah, Mahnaz Shafiei * and Aimin Yu *

School of Science, Computing, and Engineering Technology, Swinburne University of Technology, Hawthorn, VIC 3122, Australia

* Correspondence: mshafiei@swin.edu.au (M.S.); aiminyu@swin.edu.au (A.Y.)

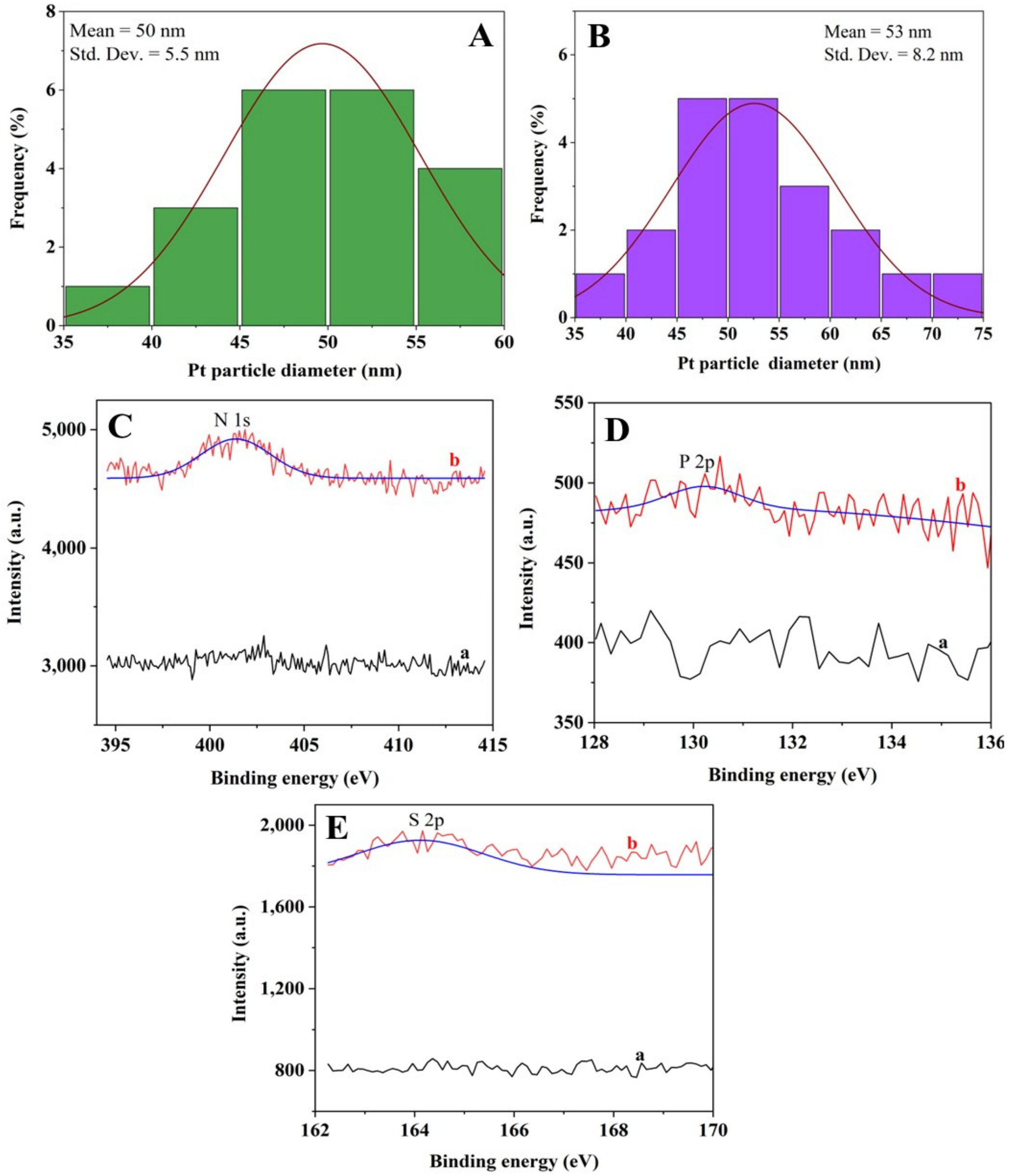

Figure S1. Pt particle size distribution histogram for **(A)** Pt-rGO/GCE, **(B)** Apt-Pt-rGO/GCE and **(C-E)** peak confirmation for N 1s, P 2p, and S 2p in Apt-Pt-rGO/GCE corresponds to XPS results.

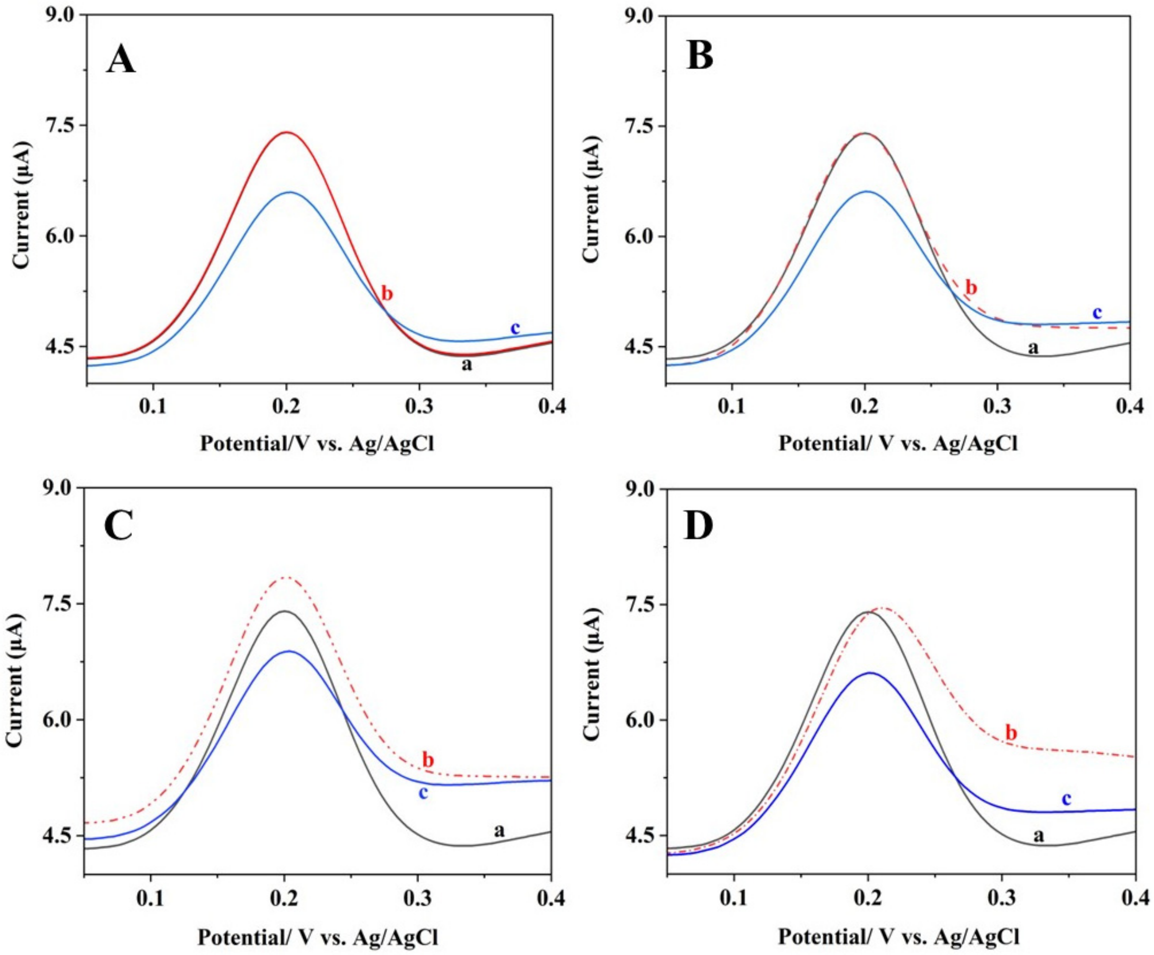

Figure S2. Regeneration of electrodes with **(A)** 10 mM NaOH **(B)** 40 mM tris-HCl (pH 8.0), 10 mM EDTA with 0.02% tween-20 **(C)** 2% SDS solution, and **(D)** 10 mM NaCl salt. Curve a indicates the current before incubation, curve b indicates the current after incubation with regeneration agents, and curve c indicates the current with 10 µM CBZ.
